# Response of *Burkholderia cenocepacia* H111 to Micro-Oxia

**DOI:** 10.1371/journal.pone.0072939

**Published:** 2013-09-02

**Authors:** Gabriella Pessi, Rubina Braunwalder, Alexander Grunau, Ulrich Omasits, Christian H. Ahrens, Leo Eberl

**Affiliations:** 1 Department of Microbiology, University of Zurich, Zürich, Switzerland; 2 Institute of Molecular Life Sciences, University of Zurich, Zürich, Switzerland; Ghent University, Belgium

## Abstract

*B. cenocepacia* is an opportunistic human pathogen that is particularly problematic for patients suffering from cystic fibrosis (CF). In the CF lung bacteria grow to high densities within the viscous mucus that is limited in oxygen. *Pseudomonas aeruginosa*, the dominant pathogen in CF patients, is known to grow and survive under oxygen-limited to anaerobic conditions by using micro-oxic respiration, denitrification and fermentative pathways. In contrast, inspection of the genome sequences of available *B. cenocepacia* strains suggested that *B. cenocepacia* is an obligate aerobic and non-fermenting bacterium. In accordance with the bioinformatics analysis we observed that *B. cenocepacia* H111 is able to grow with as little as 0.1% O_2_ but not under strictly anoxic conditions. Phenotypic analyses revealed that H111 produced larger amounts of biofilm, pellicle and proteases under micro-oxic conditions (0.5%–5% O_2_, i.e. conditions that mimic those encountered in CF lung infection), and was more resistant to several antibiotics. RNA-Seq and shotgun proteomics analyses of cultures of *B. cenocepacia* H111 grown under micro-oxic and aerobic conditions showed up-regulation of genes involved in the synthesis of the exopolysaccharide (EPS) cepacian as well as several proteases, two isocitrate lyases and other genes potentially important for life in micro-oxia.

Data deposition: RNA-Seq raw data files are accessible through the GEO Series accession number GSE48585. MS data have been deposited in the ProteomeXchange database (PXD000270).

## Introduction

*Burkholderia cenocepacia* is one of the 17 members of the *Burkholderia cepacia* complex (Bcc) whose extraordinary metabolic versatility allows it to adapt to a variety of environmental conditions, including infection sites in humans [1], [2]. Of particular concern are lung infections of patients suffering from cystic fibrosis (CF). One of the major problems associated with Bcc infections is their capacity to form highly organized surfaced-associated communities (biofilms) with an intrinsic resistance to most common antibiotics in clinical use [1], [3]. Several strains of the Bcc species *B. multivorans*, *B. cenocepacia*, *B. cepacia*, and *B. dolosa* have been shown to be highly transmissible between patients [4], with *B. cenocepacia* and *B. multivorans* accounting for the majority of CF infections [5]. During chronic colonization of the CF lung, bacteria are under strong selective pressures that result from challenges of the immune defense, antimicrobial therapy, nutrient and oxygen availability [6]. *B. cenocepacia* produces biofilms and uses the highly viscous mucus of the CF lung as a rich nutrient source. Due to bacterial respiration a steep oxygen gradient within the mucus is generated and the deeper layers become anaerobic [7]–[10]. This observation is supported by the fact that anaerobes have been found to occur in CF sputum at high cell densities [11]. Recently, Alvarez-Ortega and colleagues provided evidence that the major CF pathogen *P. aeruginosa* is growing in the CF lung preferentially by micro-oxic respiration [12]. Moreover, chemostat experiments with aerobic and micro-oxic cultures of *P. aeruginosa* suggested that this facultative anaerobe is growing optimally in a micro-oxic environment where it is producing more virulence factors such as the exopolysaccharide (EPS) alginate and pyocyanine [13]. Further studies have also shown that an anaerobic environment stimulates the production of alginate [7], [9]. In anaerobiosis, *P. aeruginosa* can utilize nitrate or nitrite rather than oxygen as a terminal electron acceptor [14]–[17]. In the absence of nitrate or nitrite, it can convert arginine to ornithine, thereby generating energy for anoxic growth [16]–[18]. Finally *P. aeruginosa* can use pyruvate fermentation for long-term survival of up to 18 days under anoxic conditions and this conversion of pyruvate into lactate, acetate, and succinate is in turn inhibited by nitrate respiration [19].

In a retrospective study of a *Burkholderia dolosa* outbreak among CF patients, the genomes of 112 isolates collected from 14 individuals over 16 years were sequenced and intriguingly revealed that 3 out of the 17 genes found to be under strong selection during pathogenesis had mutations in genes involved in oxygen-dependent regulation [20]. This suggests that sensing of a low oxygen environment is critical for pathogenesis in lung infections.

These findings posed the question of how *B. cenocepacia*, which is considered an obligate aerobe, can grow or survive in the micro-oxic/anoxic CF lung environment. Very recently, Sass and colleagues reported a low-oxygen activated locus (*lxa*) that has been shown to play an important role in regulation of the low oxygen response in *B. cenocepacia* strains J2315 and K56-2 [21]. After exposure to an anoxic environment, the *lxa* mutant showed less viable cells compared to the wild type. However, the *B. cenocepacia* H111 strain, which was originally isolated from a CF patient as well as other *B. cepacia* complex (Bcc) strains, do not possess the *lxa* locus [21], [22].

Here we show that *B. cenocepacia* H111 as well as other *B. cenocepacia* strains did not display any obvious functions that would allow anaerobic growth: no genes were found that are involved in denitrification and arginine fermentation. However, H111 is able to grow at an oxygen concentration of 0.1%, yet cannot grow anoxically in culture. When grown micro-oxically, *B. cenocepacia* produces more substratum-associated biofilm mass as well as a more robust pellicle compared to aerobic conditions. Finally, RNA-Seq and shotgun proteomics analyses from matched aerobic and micro-oxic samples were carried out to obtain a more detailed view of the repertoire of genes and proteins potentially important for growth in a low oxygen environment.

## Results

### Growth of *B. cenocepacia* H111 at different oxygen concentrations

**Figure 1:**
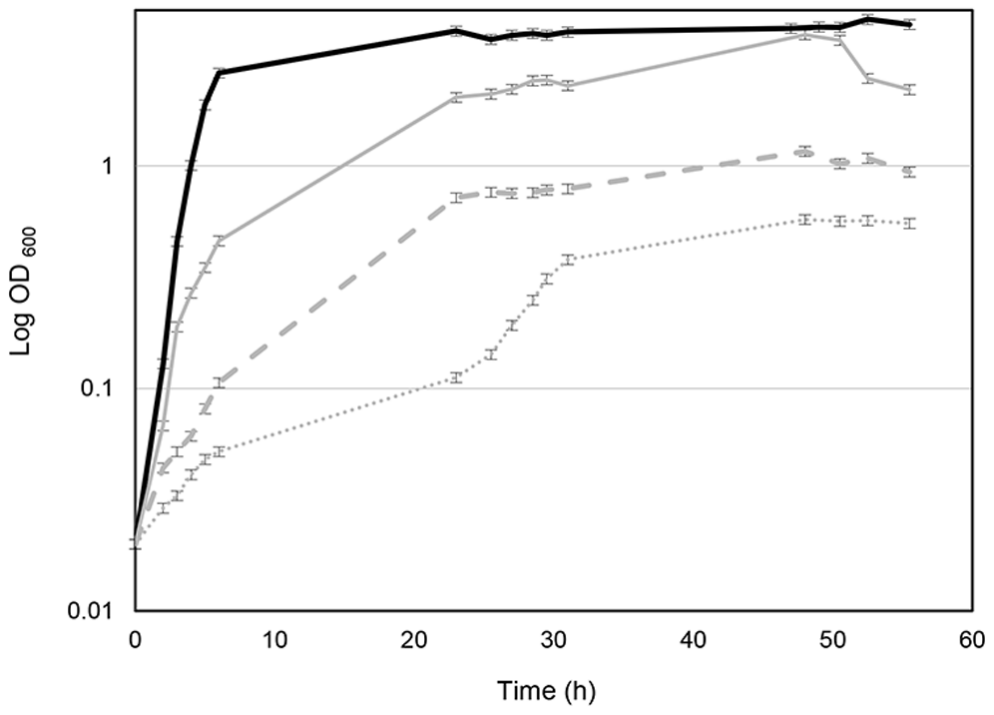
Growth of *B. cenocepacia* at different oxygen concentrations (21%, 5%, 0.5% and 0.1%). Aerobic cultures (21%, black line) were grown with shaking in 1L Erlenmeyer flasks containing 100 ml LB medium while micro-oxic cultures were grown in 500-ml rubber-stoppered serum bottles containing 25 ml LB medium in presence of a nitrogen gas atmosphere that contained 5% (grey line), 0.5% (grey dashed line) or 0.1% (grey dotted line) oxygen (Pangas). Whiskers indicate SD, n = 3.

The ability of *B. cenocepacia* to grow at different oxygen concentrations in complex media was tested. When cells were grown under normal aerobic conditions (21% O_2_), the cells grew to an optical density (OD_600_) of around 3 with a generation time of approximately 65 minutes. When only 5% or 0.5% oxygen was supplied (see Methods), the cells grew slower (generation times of 113 and 180 minutes, respectively), probably because the dissolved oxygen concentration dropped quickly to growth limiting levels (Figure 1). However, *B. cenocepacia* was still able to grow with 0.1% oxygen with a doubling time of 268 minutes, reaching an OD_600_ of 0.7. To test for growth in the absence of oxygen, several alternative electron acceptors, including nitrate, fumarate, and DMSO as well as the C-sources pyruvate, oxalate and arginine and a medium mimicking synthetic mucus [12], were tested. However, in none of the conditions tested did we observe growth in the absence of oxygen.

### Identification of genes in the *B. cenocepacia* H111 genome that may be required for growth under “low-oxygen” conditions

To identify genes related to denitrification, arginine fermentation or pyruvate fermentation, we searched for the corresponding *P. aeruginosa* orthologs in *B. cenocepacia* H111 and other sequenced *Burkholderia* species. The genes required for denitrification which encode all enzymes for nitrate/nitrite, nitric-oxide and nitrous-oxide reduction (PA3872-75, PA0509-PA0519, PA0520-24, PA3391-96), could only be identified in the “pseudomallei” group members *B. thailandensis*, *B. pseudomallei* and *B. mallei*. Indeed *B. pseudomallei* was reported to be able to survive without oxygen using nitrate respiration [23], [24]. In contrast, *B. cenocepacia* strains, including strain H111 were found to only possess the nitrite reductase encoding gene cluster (*BCAM1683*-*86*). *P. aeruginosa* is also able to generate ATP by the degradation of arginine to ornithine, which requires expression of the *arcABC* operon (PA5170-73) [18]. While *arcB* (ornithine carbamoyltransferase) is present in all sequenced *B. cenocepacia* strains, the entire operon is only present in strains of *B. thailandensis*, *B. pseudomallei*, *B. mallei*, *B. ambifaria*, *B. xenovorans*, *B. phymatum* and *B. phytofirmans*. The fermentation of pyruvate can also be used by *P. aeruginosa* to generate energy and survive during anoxic growth (PA0835-36 and PA0927) [19]. The genes necessary for the conversion of pyruvate to lactate, acetate, and succinate, i.e. the acetate kinase *ackA*, the phosphate acetyltransferase *pta* and the lactate dehydrogenase *ldhA*, were found in the genome of all *Burkholderia* species. Many bacteria adapt to micro-oxic conditions by synthetizing a particular cytochrome c oxidase (cbb3) complex with a high affinity for oxygen [25]–[27]. No classical *cbb*_3_ cytochrome oxidase was found in any of the sequenced *Burkholderia* strains. In contrast, a homolog of the *bd*-type oxidase (cyanide insensitive) was identified in the genome of several *Burkholderia* strains including strain H111 (*BCAM2674*-*75*). Homologs of the *P. aeruginosa* central regulator of anaerobic metabolism FNR/ANR (PA1544) [28] were identified in all sequenced *Burkholderia* strains. The strain H111 has two FNR/ANR orthologs, BCAM0049 and BCAM1483.

### Micro-oxic conditions favor the sessile lifestyle

**Figure 2:**
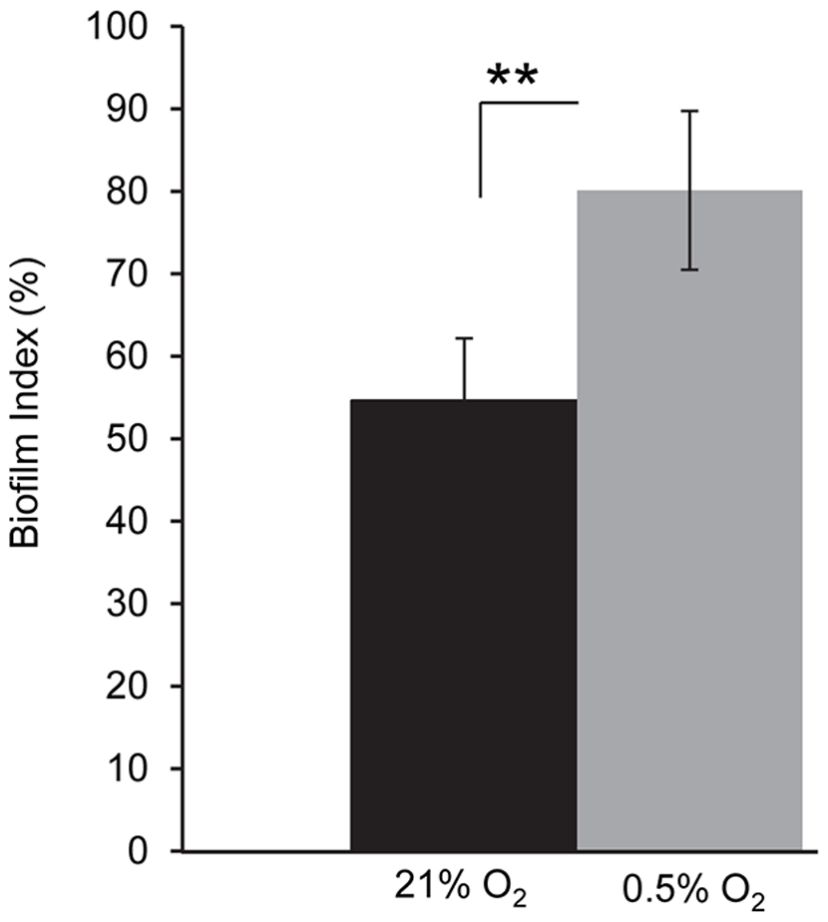
Influence of oxygen on biofilm formation in *B. cenocepacia* H111. Biofilm formation in ABC minimal medium. *B. cenocepacia* H111 was grown in 96-well plates under aerobic (black) or in micro-oxic (grey) conditions created in a CampyGen compact system (oxoid). Whiskers indicate SD, n = 3.

The capacity of our model strain H111 to form a biofilm in a polystyrene microtiter dish-based assay was tested under aerobic and micro-oxic conditions. The biofilm index (BI), i.e. biofilm mass normalized against planktonic growth, was used to compensate for the different growth rates. The amount of adhered biomass in cells grown to the begin of stationary phase was found to be significantly higher with 0.5% oxygen (Biofilm Index 80%) compared to 21% oxygen (Biofilm index 55%) (p-value<0.01, Figure 2). We also tested for pellicle formation, i.e. the biofilm formed at the liquid-air interface of static cultures and *B. cenocepacia* was found to produce more pellicle under micro-oxic conditions (data not shown). Other phenotypes such as swarming and swimming motility were not affected by oxygen availability after 48 hours of incubation. In contrast, the production of siderophores as measured on CAS plates was reduced in micro-oxically grown cells (Figure S1).

**Figure 3:**
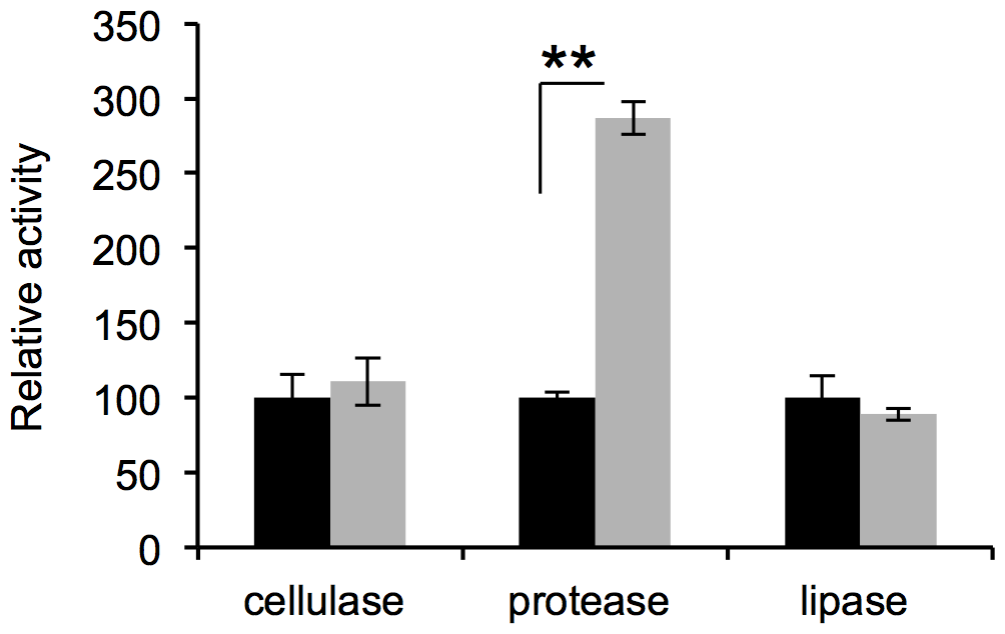
Protease activity is increased in micro-oxia. The exoenzymes cellulase, protease and lipase were measured in supernatants of aerobic (black) and micro-oxic (grey) growing cells as described in material and methods. The activity in the supernatant of aerobic cells was set to 100%. Whiskers indicate SD, n = 6.

**Figure 4:**
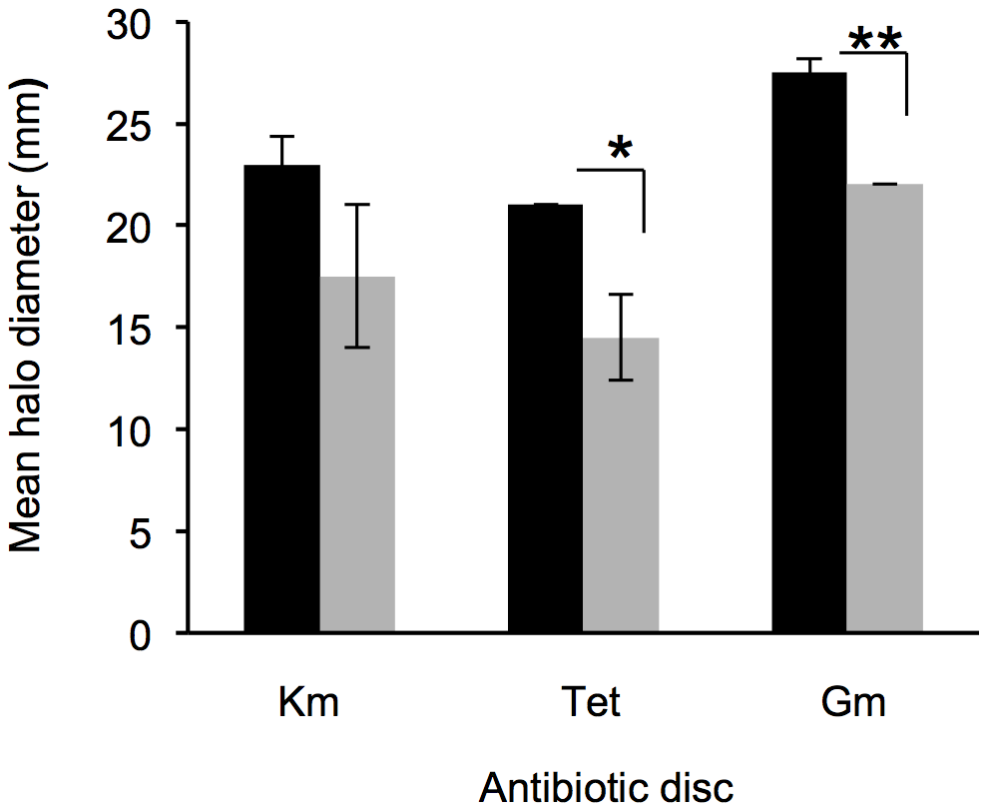
Oxygen-dependent antibiotic resistance profile of *B. cenocepacia* H111. Discs containing 30 µg Kanamycin, 30 µg Tetracycline or 10 µg Gentamycin, respectively, were placed on a plate containing *B. cenocepacia* H111 strain. Plates were incubated aerobically (black) or micro-oxically (grey) and mean halo diameters were determined. Whiskers indicate SD, n = 3.

The production of extracellular factors such as cellulases, proteases, lipases, was also investigated. These assays revealed that proteolytic activity was significantly higher under micro-oxic conditions (p-value<0.01, Figure 3) while lipolytic and cellulolytic activities remained constant and were independent of the oxygen level. To examine whether cells that were grown with low oxygen were also more resistant to antibiotics, we exposed cells grown micro-oxically and aerobically on plates to the aminoglycosides kanamycin, gentamycin and to tetracycline. Cells grown micro-oxically showed an increased resistance to all tested aminoglycosides as well as to tetracycline (Figure 4).

### Oxygen availability affects metabolic pathways

The metabolism of *B. cenocepacia* H111 grown under aerobic and micro-oxic conditions was compared using Biolog plates for carbon (C) and nitrogen (N) utilization. In these assays the strain's ability to oxidize 190 carbon and 95 nitrogen substrates was tested. An overview of all significant differences in C and N-source utilization under aerobic versus micro-oxic conditions is presented in Table 1. Interestingly, H111 was able to metabolize approximately 70% of the tested C- sources and around 90% of the investigated N-sources. We observed that micro-oxic cells grew to a 4-fold higher optical density (OD_600_) on inosine and to a 2-fold greater OD on adenosine, tricarballylic acid, malonic acid and succinamic acid compared to aerobically growing cells. For the utilization of N-sources we found a 2-fold increased respiration of ethylendiamine and D, L-α- amino-caprylic acid under micro-oxia.

**Table 1:** List of carbon- and nitrogen compounds that were differentially used in micro-oxic versus aerobic conditions.

	Aerobiosis		Micro-oxia	
Plates	3×increased	2×increased	4×increased	2×increased
	Glycyl-L-aspartic acid	α-hydroxy glutaric acid-γ lactone	Inosine	Adenosine
C-source		Propionic acid		Tricarballylic acid
		2-hydroxy benzoic acid		Malonic acid
		β-hydroxy butyric acid		2-deoxy-D-ribose
		L-Lysine		Succinamic acid
N-source		Alloxan		Ethylendiamine
		D-glucosamine		D,L-α-amino-caprylic acid
		Guanine		
		Agmatine		

Biolog plates PM1 and PM2a were used for C-source profiling and plate PM3b for N-sources utilization.

### Global transcript and protein expression changes in response to low oxygen

**Figure 5:**
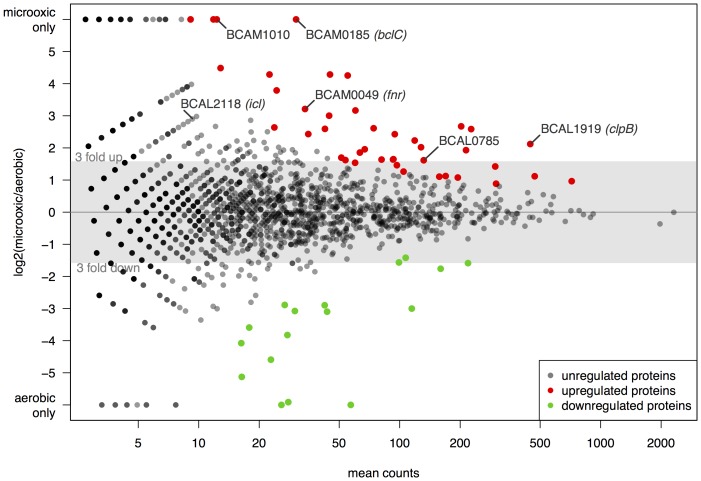
Differential protein expression under micro-oxic and aerobic conditions. MA plot showing the log2 fold change in protein expression of *B. cenocepacia* H111 grown under micro-oxic versus aerobic conditions. The top regulated proteins are shown in color: proteins with increased expression under micro-oxic conditions are indicated in red, down-regulated proteins in green.

To investigate the underlying molecular mechanisms of the observed phenotypic alterations under micro-oxic conditions we performed a transcriptomic as well as a proteomic analysis. For a global profiling of transcript and protein levels, aerobic and micro-oxic cells were grown to the late exponential phase (OD_600_ of 0.8 and 0.4, respectively, Figure 1). Total protein extracts and RNA were obtained from matched samples and further processed (see Methods). To enable detection of low abundance proteins, samples were subfractionated and analyzed using an exclusion list approach [29]. The analysis of cytoplasmic, extracellular and membrane fractions identified a total of 2128 proteins (1726 in oxia, 1911 in micro-oxia). We used DESeq [30] to generate a list of differentially expressed proteins (or genes, see below), ranked according to statistical significance (see Methods). Of the top 58 differentially expressed proteins (Figure 5) the majority (41) were up-regulated in micro-oxia. A global transcript profile analysis of the same samples identified 3806 and 4133 genes expressed aerobically and micro-oxically, respectively. Of the 123 top differentially expressed genes identified by DESeq, 102 were up-regulated in micro-oxia. Importantly, of the 58 differentially expressed proteins, 51 were also found to be similarly regulated at the transcript level. Altogether, we obtained a list of 176 genes and/or proteins that were differentially regulated by low-oxygen (Table 2). Among them, 139 genes/proteins (78%) were up-regulated in micro-oxia, including several transporters (BCAL0447, BCAS0081, BCAS0451, BCAS0602) and outer membrane proteins, genes involved in synthesis of the EPS cepacian (BCAM1004-1005 and BCAM1010), several proteases (Table 2) and an isocitrate lyase (ICL, BCAL2118). Several genes/proteins involved in reactive oxygen species (ROS) scavenging such as catalases, the alkyl hydroperoxide reductase AhpC and several thioredoxins showed increased expression in low-oxygen conditions. Among the highly up-regulated transcriptional regulators was the FNR-type regulator BCAM0049 as well as the *rpoS* homolog BCAM1259. A functional classification based on proNOG categories of the EggNOG resource [31] (see Methods) revealed that genes/proteins involved in post-translational modification, protein turnover and chaperones (category O) are over-represented in the list of genes/proteins that are up-regulated by low oxygen. In contrast, the functional categories “cell motility (category N)” and “inorganic ion transport and metabolism (category P) are enriched in the dataset of genes/proteins down-regulated in micro-oxia.

**Table 2:** List of 176 *B. cenocepacia* H111 genes/proteins that showed differential expression in micro-oxic (M) conditions compared to aerobic (A) conditions (DESeq analysis, p-value<0.15 for proteomics and p-value<0.2 for RNA-Seq).

Locus ID^a^	Orthologs J2315^b^	Description^a^	Tp^c^	Proteome FC(M/A)^d^	RNASeq FC(M/A)^e^
Amino acid transport and metabolism					
**CCE49364**	**BCAL0010**	**Phenylalanine-4-hydroxylase**		**3.2**	**1.4**
CCE53410	BCAL0705	D-alanine aminotransferase		-1.2	17.4
**CCE52708**	**BCAL2198**	**Cysteine desulfurase, IscS subfamily**		**−7.5**	**−2.4**
CCE50178	BCAL2213	Oligopeptidase A		1.5	11.7
***CCE48700***	***BCAM1111***	***Ornithine decarboxylase***		***6.1***	***2.5***
***CCE48699***	***BCAM1112***	***Arginine decarboxylase/Ornithine decarboxylase***		***6.0***	***2.2***
CCE47458	BCAM1306	Amino acid permease	TM	nd	16.6
*CCE47406*	*BCAM1353*	*Alanine dehydrogenase*		*nd*	*M only*
**CCE46974**	**BCAM1735**	**Glucose dehydrogenase, membrane-bound,flavoprotein**	**TM**	**−60.3**	**−1.4**
CCE53212	BCAM2094	Glutamine synthetase family protein		nd	15.1
CCE47595	BCAM2482	Agmatinase		nd	19.8
CCE51862	BCAS0081	ABC transporter		nd	27.9
CCE52306	BCAS0451	ABC transporter ATP-binding protein		nd	19.4
CCE52596	BCAS0602	Permease of the metabolite transporter (DMT) superfamily	TM	nd	M only
Energy production and conversion					
*CCE49315*	*BCAL0052*	*D-2-hydroxyglutarate dehydrogenase*		*nd*	*15.0*
*CCE48192*	*BCAL0522*	*Flagellum-specific ATP synthase FliI*		*nd*	*A only*
**CCE48177**	**BCAL0536**	**Ferredoxin–NADP(+) reductase**		**3.1**	**1.4**
***CCE53334***	***BCAL0785***	***Cytochrome d ubiquinol oxidase subunit I***	***TM***	***3.1***	***6.7***
**CCE50746**	**BCAL1831**	**Aldehyde dehydrogenase**		**3.1**	**1.3**
*CCE52795*	*BCAL2118*	*Isocitrate lyase*		*7.5*	*24.9*
**CCE49032**	**BCAL2685**	**Sulfite reductase [NADPH] hemoprotein β-component**		**−16.9**	**−1.5**
CCE51244	BCAL3285	Flavohemoprotein		nd	51.7
**CCE46730**	**BCAM0175**	**Malate:quinone oxidoreductase**	**S**	**−8.4**	**−3.6**
**CCE47517**	**BCAM1250**	**Acetyl-CoA hydrolase**		**9.0**	**5.5**
**CCE47209**	**BCAM1537**	**Putative oxidoreductase YncB**		**6.0**	**9.4**
***CCE47172***	***BCAM1570***	***Alcohol dehydrogenase***		***4.7***	***7.6***
***CCE47153***	***BCAM1581***	***Phosphoenolpyruvate carboxykinase [GTP]***		***8.0***	***5.6***
***CCE46975***	***BCAM1734***	***Glucose dehydrogenase***	***S***	***−7.4***	***−8.3***
*CCE53213*	*BCAM2093*	*Salicylate hydroxylase*		*nd*	*28.5*
**CCE46457**	**BCAM2710**	**Protein acetyltransferase**		**19.5**	**1.9**
CCE51861	BCAS0080	FAD-dependent NAD(P)-disulphide oxidoreductase		nd	28.1
Nucleotide transport and metabolism					
CCE47622	BCAM2458	Adenosine deaminase		nd	31.2
CCE48624	BCAM0402	Cytidine/deoxycytidylate deaminase family protein		nd	20.8
Carbohydrate transport and metabolism					
CCE51300	BCAL3342	Phosphoglycerate mutase		1.8	−19.1
CCE46772	BCAM0154	4-deoxy-L-threo-5-hexosulose-uronate ketol-isomerase		nd	16.9
Coenzyme transport and metabolism					
*CCE49767*	*BCAL2975*	*Periplasmic molybdate-binding domain protein*		*nd*	*17.3*
**CCE49194**	**BCAM0010**	**2-amino-3-ketobutyrate coenzyme A ligase**		**2.9**	**1.7**
Lipid transport and metabolism					
**CCE49544**	**BCAL1863**	**Polyhydroxyalkanoic acid synthase**		**6.2**	**1.4**
*CCE48735*	*BCAM1005*	*O-antigen acetylase*	*TM*	*nd*	*M only*
CCE53016	BCAM2232	2,3-dihydroxybenzoate-AMP ligase siderophore		nd	−38.2
Translation, ribosomal structure and biogenesis					
CCE49086	BCAL0231	Translation elongation factor G		1.4	9.9
Transcription					
*CCE49231*	*BCAL0124*	*Flagellar transcriptional activator FlhD*		*nd*	*−19.1*
**CCE48084**	**BCAL0625**	**Transcriptional regulator**		**M only**	**1.6**
CCE51487	BCAL1210	Transcriptional regulators, LysR family		nd	14.8
***CCE46853***	***BCAM0049***	***Transcriptional regulator, CRP family***		***9.3***	***3.8***
CCE46759	BCAM0167	Transcriptional regulator, LysR family		nd	21.4
CCE48623	BCAM0403	Acetyltransferase		nd	22.9
CCE48936	BCAM0751	Transcriptional regulator, LysR family		nd	11.7
CCE47511	BCAM1257	Transcriptional regulator, MerR family		nd	M only
*CCE47508*	*BCAM1259*	*RpoD-related RND polymerase sigma factor*		*nd*	*26.4*
CCE47210	BCAM1536	Transcriptional regulator, TetR family		1.7	20.7
CCE52597	BCAS0603	Transcriptional regulator, AraC family		nd	28.1
CCE53209		Transcriptional regulator, TetR family		nd	14.6
Replication, recombination and repair					
CCE52866	BCAL2278	Transposase		nd	9.7
CCE47509	BCAM1258	Putative DNA polymerase family X		nd	18.5
Cell wall/membrane/envelope biogenesis					
*CCE50995*	*BCAL0940*	*Membrane carboxypeptidase (penicillin-binding protein)*	*TM*	*nd*	*11.0*
**CCE51437**	**BCAL1258**	**Membrane-bound murein transglycosylase D precursor**		**−35.0**	**1.5**
**CCE47918**	**BCAL1493**	**Putative transmembrane protein**		**−3**	**−1.5**
***CCE50748***	***BCAL1829***	***Outer membrane protein W precursor***	***S***	***6.4***	***3.8***
***CCE50709***	***BCAL2645***	***Outer membrane protein***	***TM***	***3.1***	***−1.2***
***CCE49628***	***BCAL2783***	***Cyclopropane-fatty-acyl-phospholipid synthase***		***5.4***	***3.6***
**CCE49806**	**BCAL3008**	**Outer membrane protein (porin)**	**S**	**2.1**	**2.1**
**CCE51186**	**BCAL3204**	**Peptidoglycan-associated lipoprotein precursor**	**S**	**−2.7**	**−1.3**
*CCE48736*	*BCAM1004*	*GDP-mannose 4,6 dehydratase*		*M only*	*12.2*
**CCE48728**	**BCAM1010**	**UTP–glucose-1-phosphate uridylyltransferase**		**M only**	**2.7**
**CCE47356**	**BCAM1398**	**Outer membrane protein (porin)**	**S**	**−3.4**	**−4.1**
CCE46444	BCAM2723	Outer membrane porin, OprD family	S	nd	20.1
Cell motility					
*CCE51168*	*BCAL0142*	*Flagellar biosynthesis protein FlhF*		*4.4*	*−44.9*
*CCE48144*	*BCAL0567*	*Flagellar hook protein FlgE*		*−2.2*	*−10.8*
*CCE48143*	*BCAL0568*	*Flagellar basal-body rod protein FlgF*		*nd*	*−20.2*
*CCE48141*	*BCAL0570*	*Flagellar L-ring protein FlgH*	*S*	*−2.1*	*−20.8*
*CCE48139*	*BCAL0572*	*Flagellar protein FlgJ [peptidoglycan hydrolase]*		*nd*	*−24.7*
*CCE47986*	*BCAL3503*	*Flagellar biosynthesis protein FliP*	*TM*	*nd*	*−19.5*
CCE47983	BCAL3506	Flagellar motor switch protein FliM		−1.4	−20.7
**CCE53442**	**BCAL1677**	**Type 1 fimbriae major subunit FimA**	**S**	**1.8**	**M only**
Posttranslational modification, protein turnover, chaperones					
CCE48216	BCAL0500	ATP-dependent hsl protease ATP-binding subunit HslU		1.3	13.3
CCE51547	BCAL1070	Alkyl hydroperoxide reductase subunit C-like protein	S	1.0	15.0
*CCE51462*	*BCAL1233*	*Molecular chaperone (small heat shock protein)*		*2.1*	*32.5*
*CCE51461*	*BCAL1234*	*Molecular chaperone (small heat shock protein)*		*5.3*	*37.4*
***CCE49486***	***BCAL1919***	***ClpB protein***		***4.3***	***20.9***
**CCE49077**	**BCAL2730**	**ATP-dependent protease ATP-binding subunit ClpA**		**3.9**	**7.2**
CCE49078	BCAL2731	ATP-dependent Clp protease adaptor protein ClpS		2.5	11.0
*CCE49625*	*BCAL2780*	*Thioredoxin domain-containing protein EC-YbbN*		*1.7*	*10.6*
**CCE52629**	**BCAL3146**	**Heat shock protein 60 family chaperone GroEL**		**2.7**	**6.7**
CCE51225	BCAL3269	Chaperone protein DnaJ		1.7	12.2
**CCE51226**	**BCAL3270**	**Chaperone protein DnaK**		**2.0**	**8.9**
CCE51227	BCAL3271	Thiol-disulfide isomerase and thioredoxins		nd	14.3
CCE51228	BCAL3272	Heat shock protein GrpE		1.1	9.7
CCE47165	BCAM0309	Cell division protein FtsH	TM	nd	17.5
CCE48963	BCAM0727	Membrane protease subunits, stomatin/prohibitin homologs		nd	68.6
CCE46962	BCAM1744	Extracellular protease precursor	S	1.3	−18.2
CCE52630	BCAS0638	Heat shock protein 60 family co-chaperone GroES		2.2	27.6
CCE52633	BCAS0641	serine protease		nd	56.1
Inorganic ion transport and metabolism					
CCE49312	BCAL0055	Copper-translocating P-type ATPase	TM	1.5	11.5
CCE52829	BCAL0447	Ferric iron ABC transporter, permease protein	TM	nd	M only
CCE51464	BCAL1231	Integral membrane protein TerC	TM	nd	M only
**CCE49029**	**BCAL2682**	**Sulfate adenylyltransferase subunit 2**		**−14.2**	**−2.5**
**CCE51255**	**BCAL3299**	**Catalase/Peroxidase**		**2.4**	**1.9**
***CCE48540***	***BCAM0491***	***Outer membrane vitamin B12 receptor BtuB***	***S***	***−24.1***	***−2.1***
**CCE48794**	**BCAM0948**	**Outer membrane protein NosA precursor**		**−8.0**	**−14.2**
***CCE47584***	***BCAM1187***	***Ferrichrome-iron receptor***		***A only***	***−10.9***
*CCE47171*	*BCAM1571*	*Zinc-regulated outer membrane receptor*		*M only*	*37.1*
***CCE49127***	***BCAM2007***	***Ferrichrome-iron receptor***	***S***	***A only***	***−13.8***
CCE53024	BCAM2224	Outer membrane receptor for ferric-pyochelin FptA	S	nd	−20.2
***CCE47643***	***BCAM2439***	***Ferrichrome-iron receptor***	***S***	***−3.0***	***−7.3***
CCE52627	BCAS0635	Manganese catalase		nd	M only
Secondary metabolites biosynthesis, transport and catabolism					
CCE53019	BCAM2230	Dihydroaeruginoate synthetase PchE		nd	−14.3
CCE53020		Pyochelin synthetase PchF		nd	−27.3
Signal transduction mechanisms					
***CCE53457***	***BCAL1663***	***Serine protein kinase (PrkA protein)***		***19.5***	***16.5***
***CCE52508***	***BCAM0276***	***Universal stress protein UspA***		***4.1***	***14.1***
*CCE50874*	*BCAM0877*	*Diadenosine tetraphosphatase*		*nd*	*M only*
CCE46268	BCAM2563	Aerotaxis sensor receptor protein	TM	nd	10.3
Intracellular trafficking, secretion, and vesicular transport					
***CCE47881***	***BCAL1529***	***Type II/IV secretion system ATPase TadZ***		***22.4***	***1.5***
CCE53120	BCAM2140	HlyD family secretion protein	TM	nd	30.7
Others					
CCE49314	BCAL0053	Transcriptional regulator, PadR family		1.1	12.3
CCE51109	BCAL0213	Phenylacetate-CoA oxygenase, PaaJ subunit		nd	−34.8
CCE51108	BCAL0214	Phenylacetate-CoA oxygenase, PaaI subunit		2.9	−10.4
**CCE46576**	**BCAL0342**	**Uncharacterized protein ImpC**		**2.2**	**−1.8**
**CCE46575**	**BCAL0343**	**Uncharacterized protein ImpD**		**2.2**	**−1.0**
*CCE48020*	*BCAL0683*	*Hypothetical protein I35_1851*		*nd*	*20.6*
*CCE53333*	*BCAL0786*	*Hypothetical protein I35_7268*	*TM*	*nd*	*10.0*
CCE51079	BCAL0860	Staphylolytic protease preproenzyme LasA		nd	14.3
CCE50996	BCAL0939	Gfa-like protein		nd	12.2
CCE51463	BCAL1232	Hypothetical protein I35_5360		nd	M only
**CCE51401**	**BCAL1294**	**VgrG protein**		**13.8**	**2.6**
CCE51631	BCAL1463	Ribonuclease BN	TM	nd	21.7
*CCE53456*	*BCAL1664*	*Hypothetical protein I35_7395*		*nd*	*16.7*
*CCE53455*	*BCAL1665*	*SpoVR-like protein*		*nd*	*16.4*
***CCE50747***	***BCAL1830***	***Dioxygenase,2-nitropropane dioxygenase-like***		***19.1***	***1.8***
CCE50719	BCAL1857	Hypothetical protein I35_4602	TM	nd	16.0
**CCE52700**	**BCAL2206**	**Granule-associated protein**		**3.1**	**9.5**
CCE50568	BCAL2439	Hypothetical protein I35_4448	TM	nd	12.9
CCE49604	BCAL2760	UPF0434 protein YcaR		−3.0	A only
**CCE49988**	**BCAL3178**	**Transcriptional regulator**		**2.8**	**1.7**
***CCE51204***	***BCAL3243***	***Capsular polysaccharide biosynthesis/export protein***		***−8.6***	***2.2***
*CCE46874*	*BCAM0028*	*Hypothetical protein I35_0684*		*nd*	*31.2*
*CCE46761*	*BCAM0165*	*Hypothetical protein I35_0571*		*nd*	*−14.1*
**CCE46721**	**BCAM0185**	**Lectin BclC**		**M only**	**7.1**
CCE48935	BCAM0752	Hydrolase-related protein		nd	15.2
CCE47460	BCAM1304	Phage-related protein		nd	10.6
CCE47459	BCAM1305	hypothetical protein I35_1271		nd	10.8
*CCE47408*	*BCAM1351*	*DnaK suppressor protein*		*nd*	*16.2*
*CCE47407*	*BCAM1352*	*DNA-dependent DNA polymerase family X*		*nd*	*27.2*
*CCE47250*	*BCAM1500*	*Universal stress protein family*		*5.4*	*5.6*
CCE47213	BCAM1534	Chromosome segregation ATPases		nd	43.6
CCE47212	BCAM1535	Hypothetical protein I35_1024	S	nd	18.0
*CCE47173*	*BCAM1569*	*Neuraminidase (sialidase)*	*S*	*nd*	*13.9*
*CCE50317*	*BCAM1926*	*CBS domain protein*		*1.2*	*10.2*
CCE53123	BCAM2137	Transcriptional regulatory protein		nd	20.0
CCE53121	BCAM2139	Eukaryotic putative RNA-binding region RNP-1 signature		nd	M only
*CCE53091*	*BCAM2167*	*Hypothetical protein I35_7022*		*nd*	*15.6*
CCE53041	BCAM2210	Hypothetical protein I35_6972	TM	nd	M only
CCE47618	BCAM2462	Outer membrane protein (porin)	S	nd	55.2
CCE47617	BCAM2463	Hypothetical protein I35_1430		nd	10.2
CCE51782	BCAS0002	Chromosome (plasmid) partitioning protein ParB		1.3	19.1
CCE51864	BCAS0082	Hydrolases of the alpha/beta superfamily	TM	nd	21.1
*CCE52109*	*BCAS0293*	*AidA*		*2.2*	*M only*
CCE52595	BCAS0601	Putative ATP/GTP-binding protein		nd	28.5
CCE52677	BCAS0723	Putative cytoplasmic protein		nd	30.7
**CCE46207**		**Outer membrane protein (porin)**	**S**	**2.2**	**2.0**
CCE46671		Hypothetical protein I35_0480		nd	−9.6
CCE47170		Hypothetical protein I35_0982		nd	52.5
CCE47794		Hypothetical protein I35_1612		nd	M only
CCE48729		Hypothetical protein I35_2566	TM	nd	M only
CCE50639		Shufflon-specific DND recombinase		nd	10.6
**CCE51201**		**Capsular polysaccharide export system protein KpsE**	**TM**	**−12.1**	**1.2**
CCE52058		Quinone oxidoreductase (NADPH:quinone reductase)		nd	14.0
CCE52231		Histone acetyltransferase HPA2		nd	M only
**CCE52465**		**29 kDa antigen**		**3.6**	**−2.3**
**CCE52505**		**Regulator of competence-specific genes**		**M only**	**15.4**
CCE52619		TPR repeat protein, SEL1 subfamily	S	nd	17.1
CCE52635		Hypothetical protein I35_6546		nd	16.3
CCE52659		Tannase precursor		nd	24.9
CCE52669		Hypothetical protein I35_6580		nd	23.4
CCE53181		Cyclohexanone monooxygenase		nd	31.2
**CCE53450**		**Large exoproteins involved in heme utilization**		**3.8**	**1.9**

a Nomenclature and description according to GenBank file CAFQ01000001.1.

b Orthologs were identified as described in the Material and Methods section.

c Predicted topology (Tp) according to SignalP v4.0 (secreted proteins, S) and TMHMM v2.0 (transmembrane, TM).

d Fold change (FC) of protein expression, comparing micro-oxically (M) with aerobically (A) grown wild-type strain.

e Fold change (FC) of transcript expression, comparing micro-oxically (M) with aerobically (A) grown wild-type strain.

nd: The gene was not identified on protein level.

M only and A only: The gene/protein was detected only micro-oxically (M) or aerobically (A).

The proNOG categories are indicated and the 58 differentially expressed proteins are indicated in bold. The overlap in low oxygen regulation with strain J2315 (Sass et al., 2013) is indicated in italics.

To further validate the global analysis data, the up-regulation of several genes was confirmed by qPCR (Table S3). These included up-regulation of *BCAM0049* and *BCAM1259* expression as well as increased expression of the protease gene *BCAL1919* (*clpB*), the cytochrome d ubiquinol kinase gene *BCAL0785*, the sugar transferase gene involved in cepacian synthesis (*BCAM1010*) and the ICL encoding gene (*BCAL2118*). In addition, transcriptional *lacZ* fusions to promoter regions of selected genes up-regulated in micro-oxic conditions were constructed and measured (Figure S2). The promoter of a gene involved in cepacian biosynthesis (sugar transferase *wcaJ*), the thioredoxin *BCAL2780*, the *rpoS* homolog (*BCAM1259*) and the lectin encoding gene *BCAM0185* showed increased activity when cells were grown with low oxygen (Figure S2). As a control we used P*_cepI_*-*lacZ* transcriptional fusion and confirmed that the expression of the AHL encoding gene *cepI* was not affected by oxygen availability (confirming our RNA-Seq data).

## Discussion

At present very little is known of how *B. cenocepacia* strains can adapt to the micro-oxic/anoxic environment within biofilms in the CF lung [7]–[9], [32]. While the CF pathogen *P. aeruginosa* uses denitrification and fermentation of arginine to generate energy for growth and survival in an environment depleted of oxygen [16], [17], we could only detect very few orthologs of the respective genes involved in these processes in the genomes of *B. cenocepacia* strains. Denitrification genes were exclusively found in the genomes of members of the “pseudomallei” group, namely *B. thailandensis*, *B. pseudomallei* and *B. mallei*. Only strains of *B. thailandensis*, *B. pseudomallei*, *B. mallei*, *B. ambifaria*, *B. xenovorans*, *B. phymatum* and *B. phytofirmans*, harbor genes that potentially allow these species to ferment arginine to gain energy (1 mol of ATP per mol of arginine). In accordance with these findings it has been reported that the diversity of *Burkholderia* strains growing under anoxic conditions in soils is very low [33].

The facultative intracellular pathogen *Mycobacterium tuberculosis* has recently been shown to adapt to and recover from hypoxia using isocitrate lyase (ICL)-mediated production of succinate [34]. ICL is a glyoxylate shunt enzyme, which generates succinate whose secretion was proposed to help maintain membrane potential and ATP synthesis. The produced succinate is also a substrate of the succinate dehydrogenase (SDH) in the TCA cycle which is important for the electron transport chain by coupling carbon flow to ATP synthesis [35]. The gene encoding ICL has been shown to be up-regulated in persister cells in *B. cenocepacia* biofilms [36]. The authors of this study suggested that surviving persister cells downregulate the TCA cycle to avoid production of ROS and at the same time activate an alternative pathway, the glyoxylate shunt. Employing a combined RNA-Seq and proteomics approach we found that the two ICL genes present in the H111 genome as well as genes/proteins involved in ROS scavenging such as catalases, AhpC and several thioredoxins were up-regulated by low oxygen. Given that the same genes were also up-regulated in micro-oxia in strain J2315 [21], it is tempting to speculate that this pathway is used by *B. cenocepacia* to sustain production of ATP under micro-oxic conditions.

A phenotypical characterization revealed that micro-oxic cells grew better with purines as C-source. The up-regulation of two adenosine deaminases (Table 2 and Table S1) which are key enzymes of purine metabolism and convert adenosine to inosine suggest a role of purine metabolism in micro-oxia. Interestingly, a recent report on hepatocarcinoma-derived cells showed that purines such as inosine and adenosine have a cytoprotective effect and can serve as an alternative source of energy to produce ATP during hypoxic conditions [37]. The ribose moiety of adenosine and purine could be used as a precursor for the phosphorylated glycolytic intermediates in reactions catalyzed by the pentose phosphate (PP) pathway. Among the genes up-regulated in micro-oxia (Table S1) we also found several nucleoside phosphorylases which catalyse the reversible phosphorolysis of purine (2′-deoxy)ribonucleosides to free bases and (2′-deoxy)ribose 1-phosphates. This could represent another possibility for *B. cenocepacia* to generate energy under micro-oxic conditions.

In this study we showed that micro-oxic conditions promoted biofilm formation of *B. cenocepacia*. Similar observations have been made for *P. aeruginosa*, which produces more alginate when oxygen is limiting [38]–[40]. Our global expression analyses revealed the up-regulation of three regions potentially responsible for increased biofilm formation under micro-oxic conditions: i) the EPS cepacian encoding gene cluster *BCAM1004-10* [41], [42] ii) the lectin gene *BCAM0185* [43], and iii) the gene encoding the large surface protein BapA, which was previously shown to be important for biofilm formation [43].

The observation that cells growing micro-oxically were more resistant to several antibiotics is probably due to their slower growth rate compared to aerobically growing cells. Muir et al. showed that the higher the growth rate of cells at the time of antibiotic addition, the greater the growth-inhibitory effect [44].

The effect of low-oxygen tension on gene expression was one of the nine conditions tested by Sass and colleagues in *B. cenocepacia* strain J2315 [21]. Although the experimental settings used in their study were very different from ours (i) shift *versus* run out experiment, ii) CampyGen Compact gas generating system *versus* controlled gas atmosphere, iii) 6% *versus* 0.5% oxygen, iv) strain J2315 *versus* H111) and different analysis technologies were used (microarray *versus* RNA-Seq), there was a good overlap between the two data sets. In fact, 55 of the 176 H111 genes/proteins reported here were also differentially expressed in response to low oxygen in strain J2315 (Table S2). Among them are universal stress proteins, the protease ClpB, the isocitrate lyase BCAL2118, arginine/ornithine decarboxylases, the cytochrome d ubiquinol oxidase and several membrane proteins. In line with the observation that strain H111 produces reduced amounts of siderophores in micro-oxia, several TonB dependent receptors were down-regulated in micro-oxic conditions. The *lxa* locus as well as the cable pilus cluster (*cbl*), which are both induced in strain J2315, are absent in strain H111. Other gene clusters for flagellar and chemotaxis proteins were up-regulated only in strain J2315. Among the genes specifically induced in strain H111 we found the fimbriae encoding gene *fimA* (*BCAL1677*), an adenosine deaminase (*BCAM2458*), several porins (*BCAM2723*, *BCAL3007*, *BCAM2462*) and several ABC transporters. Among the transcriptional regulators highly up-regulated in micro-oxia in both studies was the FNR-type regulator BCAM0049 (Table 2). Orthologous proteins have been shown to sense the oxygen tension and control gene expression under low oxygen conditions in several organisms [28], [45]. The *P. aeruginosa* FNR-type regulator ANR is known to positively control expression of denitrification and arginine fermentation genes. This regulator could also play an important role in the regulation of genes in micro-oxic conditions.

In conclusion, we have shown that *B. cenocepacia* H111 can grow with as little as 0.1% oxygen but is not able to grow anaerobically. Since *P. aeruginosa* grows anaerobically and has been shown to occupy deeper sites within wounds [46] it appears likely that *B. cenocepacia* may occupy a different niche where oxygen is limited but not totally absent. Our study provides a list of the most significant differentially expressed genes/proteins in micro-oxically versus aerobically grown cells and opens new avenues in the understanding of the molecular mechanism underlying the physiology and regulation of the *in vivo* relevant micro-oxic lifestyle of *B. cenocepacia.*

## Materials and Methods

### Bacterial strains, plasmids and growth conditions

*B. cenocepacia* wild type H111 [22], [47], [48] was grown under aerobic (21% oxygen) and micro-oxic conditions (0.1% to 5% oxygen) at 37°C in LB Lennox broth (Difco) or ABC Minimal Medium containing citrate as carbon source [49]. Aerobic cultures were grown with rigorous shaking (220 rpm) in 500-mL Erlenmeyer flasks containing 25 ml medium or, for RNA-Seq and proteomics experiments, in 1-L Erlenmeyer flasks containing 100 ml medium. Micro-oxic liquid cultures were grown under a nitrogen gas atmosphere that contained 5% or 0.5% or 0.1% oxygen with moderate shaking (80 rpm) in 500-ml rubber-stoppered serum bottles containing 50 ml medium. The gas phase (e. g 0.5% O_2_, 99.5% N_2_) was exchanged every 8–14 hours. For the cultivation of bacteria on plates, micro-oxic conditions were created using the CampyGen Compact gas generating system (oxoid) by quickly changing the paper sachet every 24 hours and keeping the exposure to atmospheric oxygen at a minimum.

### Phenotypical analysis

Biofilm formation was quantified in a microtiter dish assay as described by Huber *et al*. [50]. Since micro-oxic and aerobic cells reached different optical densities (OD), we used the biofilm index (BI) to compare the amounts of biofilm formed. The BI was calculated as the mean percentage ratio between OD_570_ after crystal violet staining and OD_550_ measured before incubating the cells with crystal violet which reflects the total cell number [51]. The formation of pellicles was assessed in NYG medium (0.5% peptone, 0.3% yeast extract, 2% glycerol) according to Fazli et al., 2011 [52]. Proteolytic activity was quantified based on the method described by Schmid et al [53] growing cells in NYG medium at 37°C to late exponential growth phase and using azocasein (5 mg/ml, in 50 mM Tris-Cl pH 8) for 60 min at 37°C as substrate. For quantification of lipases and cellulases, the sterile culture supernatant was incubated with buffer 1 (1 volume 0.3% p-nitrophenyl palmitate in isopropanol and 9 volumes of 0.2% sodiumdesoxycholate and 0.1% gum arabicum in 50 mM sodiumphosphate buffer pH 8) and 1% carboxymethylcellulose, respectively. After incubation, the absorbance was measured at 410 nm and 575 nm, respectively [50]. A Bradford assay (Coomassie Plus™, Thermo Scientific/Pierce) with BSA as standard was used to determine the total protein concentration in extracts derived from both aerobic and micro-oxic cultures. Antibiotic susceptibility testing was performed on agar plates where bacteria were homogeneously spread over the surface of the agar plate. Antibiotic discs (kanamycin 30 µg, tetracycline 30 µg, gentamycin 10 µg; Alere GmbH) were placed in the center of the plate. Swarming and swimming were tested by inoculating cells onto plates containing ABC medium supplemented with 0.1% casamino acids that were solidified with 0.4% and 0.3% agar, respectively. Plates were incubated for 2 days. Siderophores production was measured on CAS plates as described previously [54]. All phenotypic assays were performed at least in triplicate.

### Biolog analysis

*B. cenocepacia* was streaked on R2A agar plates and grown overnight at 37°C. From this plate, colonies were picked up and suspended in the GN/GP-IF at the required optical density. The suspensions were then inoculated on Biolog plates PM1 and PM2a for the carbon sources and PM3b for the nitrogen sources (Biolog, Hayward, CA). Plates were incubated at 37°C for 24 h fully aerated or for 36 h under micro-oxic conditions using CampyGen jars (Oxoid, Basingstoke, UK). The optical density was measured using a plate reader; instances where a >50% OD_600_ difference was observed between micro-oxic and aerobic cells were deemed significant [55]. Each condition was tested in triplicate.

### RNA-Seq and data analysis

Total RNA from *B. cenocepacia* strain H111 grown with 21% or 0.5% oxygen in complex LB medium to the end of the exponential phase (OD_600_ of 0.8 and 0.4, respectively, Figure 1) was extracted using a modified hot acid phenol protocol [56]. The removal of genomic DNA using DNAseI (Promega) was verified by a PCR reaction with 40 cycles. The samples were then further purified using the RNeasy kit (Qiagen) and the RNA quality was checked using RNA Nano Chips (Agilent 2100 Bioanalyzer; RIN >8). The RNA samples were poly(A)-tailed using poly(A) polymerase. Then, the 5′PPP were removed using tobacco acid pyrophosphatase (TAP). Afterwards, an RNA adapter was ligated to the 5′-monophosphate of the RNA. First-strand cDNA synthesis was performed using an oligo(dT)-adapter primer and the M-MLV reverse transcriptase (Promega). The resulting cDNA was PCR-amplified to about 20–30 ng/µl using a high fidelity DNA polymerase. The cDNA was purified using the Agencourt AMPure XP kit (Beckman Coulter Genomics) and was analyzed by capillary electrophoresis. The primers used for PCR amplification were designed for TruSeq sequencing according to the instructions of Illumina. Illumina single-end sequencing was performed on a HiSeq2000 instrument. The sequence reads were processed and then mapped to the *B. cenocepacia* H111 genome using CLC Genomics Workbench v4.9 (CLC bio) allowing up to 2 mismatches per read. The mapped reads (or spectral counts, see below) were analyzed using the DESeq software [30]. DESeq models gene/protein expression with a negative binomial distribution and outputs a list of differentially expressed genes/proteins ranked according to statistical significance. We report the top 123 differentially expressed genes (p-value cut-off <0.2), i.e. approx. 2,5% of the genes found actively expressed. This model is more robust against over-identifying candidate regulated genes based on fold-change alone, which can in particular be problematic for genes that are identified with few sequencing reads (common for *Burkholderia* with their high GC content, [57]) or spectra. We only considered genes with five or more reads for differential analysis. For functional annotation of H111 genes, we relied on the eggNOG resource [31] and transferred the functional annotations from the respective J2315 orthologs as described [53]. The RNA-Seq raw data files are accessible through the GEO Series accession number GSE48585.

### Preparation of protein samples

Extracellular proteins and subcellular fractions were prepared as described previously [53]. Cells were lysed by two consecutive passes through a French Press homogenizer (Hypramag/Aminco), and cell debris was removed by 15 min centrifugation at 4000 g. Total cell membranes were subsequently harvested by ultracentrifugation for 1 h at 80000 g, 4°C. The pellet containing total membrane proteins was dissolved in 100 mM Tris-HCl, pH 7.5, 2% SDS by incubation at 50°C for 1 h. The cell lysate supernatant containing soluble cytoplasmic proteins was extracted with 6 volumes of ice-cold acetone at −20°C overnight. The precipitated proteins were harvested by centrifugation at 20000 g and dissolved in 100 mM Tris-HCl, pH 7.5, 0.1% SDS. Total protein concentration was determined according to Bradford (Coomassie Plus™ protein assay, Pierce). Approximately 15 mg total protein for each extracellular (EC), cytoplasmic (Cyt) and total membrane (TM) fractions were separated by 1D SDS-PAGE on 12.5% polyacrylamide gels. Gels were stained with colloidal Coomassie Blue (Serva). Individual protein lanes were cut into ten slices and immediately subjected to in-gel tryptic digestion [58].

### Mass spectrometry, protein identification and differential expression analysis

Peptides were separated by RP-HPLC and analyzed by a hybrid LTQ-Orbitrap XL mass spectrometer (Thermo Fisher Scientific, Waltham, MA, USA) interfaced with a nanoelectrospray source. Mass spectrometric detection was performed in data-dependent mode. Precursor mass spectra were acquired at the Orbitrap mass analyzer with a scan range from m/z 300 to 1,600; resolution was set to 60,000 at m/z 400. Mass spectra were processed with Xcalibur 2.0.7 (Thermo Fisher Scientific) and peak lists were generated with msConvert (version 3.0.4388) [59]. Fragment ion mass spectra were searched with MS-GF+ (MS-GFDB v7747) against a sequence database consisting of 7,258 *B. cenocepacia* strain H111 proteins (accession CAFQ00000000.1) and 259 common contaminants (e.g. human keratin, trypsin). Spectra were searched for a match to fully-tryptic and semi-tryptic peptides with a mass tolerance of 10ppm. Carbamidomethylation was set as fixed modification for all cysteines while oxidation of methionines, deamidation of asparagines and glutamines as well as cyclization of N-terminal glutamines were considered as optional modifications.

Based on the target-decoy search strategy [60], a stringent score cutoff was determined that resulted in an estimated FDR of less than 0.2% at the PSM level. PSMs above this cutoff were subjected to a PeptideClassifier analysis [61] and only peptides that unambiguously identify one protein (either class 1a or 3a) were considered. We furthermore required at least 3 independent spectra or two spectra from two distinct peptides for protein identification. Each subcellular fraction was measured once with a discovery run followed by a subsequent exclusion list run (precursor ions identified in the discovery run were excluded from fragmentation in the exclusion run) [29]. Thereby, about 15% more proteins (272), all preferentially lower abundant, could be added by the exclusion list approach to those identified over all respective first runs (1854, Figure S3). This resulted in a total of 2128 identified proteins at an estimated FDR of less than 1% (0.98%). Total spectral counts for each protein were used for a differential expression analysis with the R package DESeq (version 1.6.1, [30]). Due to the lower number of spectral counts compared to sequenced reads, we chose a more lenient cut-off of p<0.15 to select the 58 top-ranked differentially expressed proteins for further analysis (roughly 2.7% of all proteins expressed). Protein abundance was estimated according to the method of Schrimpf et al. [62] (Figure S3). Proteomics data associated with this manuscript can be downloaded from the ProteomeXchange under accession number PXD000270. Signal peptide predictions from SignalP (version 4.0), and transmembrane domain predictions from TMHMM (version 2.0; both from the CBS, Denmark), were used for a combined topology prediction: Proteins without a predicted transmembrane domain after a predicted signal peptide cleavage site are considered secreted. Proteins with one or more predicted transmembrane domains after a predicted signal peptide cleavage site or without a predicted signal peptide cleavage site are assumed to be transmembrane proteins.

### Construction and assessment of transcriptional *lacZ* fusions

For construction of transcriptional *lacZ* fusions, vector pSU11p [53] was used. The promoter regions of *BCAL2780*, *BCAM1259*, *wcaJ* genes were first amplified using the primers listed in Table S4 and cloned into vector pCR 2.1 TOPO (Invitrogen, Carlsbad, CA). After sequence verification, the promoter probes were cut and cloned into pSU11p using HindIII and XhoI. The resulting plasmids pP*_BCAL2780_*-*lacZ*, pP*_BCAM1259_*-*lacZ* and pP*_wcaJ-_lacZ* were transferred by triparental mating into *B. cenocepacia* strain H111 and ß-galactosidase activity was determined both under micro-oxic and aerobic conditions by the Miller method [63]. Briefly, the strains were grown overnight in LB broth, then subcultured in LB medium and incubated for 2 days (aerobic cultures) or 4 days (micro-oxic cultures). The experiment was run in triplicate. ß-galactosidase activity was also visually inspected on LB plates containing 5-bromo-4-chloro-3-indolyl-β-D-galactoside (X-Gal) (Sigma). Bacterial strains, plasmid and primers used in this study are listed in Table S4.

### qPCR analyses

The expression of H111 orthologs of J2315 genes *BCAM1259*, *BCAL0785*, *BCAL1919*, *BCAM1010*, *BCAM0049* and *BCAL2118* was analyzed with a Mx3000P instrument using Brilliant III Ultra-Fast SYBR® Green QPCR Master Mix (Agilent, Switzerland) and cDNA prepared from biological replicates as template. Each reaction contained 12.5 µl 2× Brilliant III Ultra-Fast SYBR® Green QPCR Master Mix, 0.7 µM of individual primers and 15 or 7.5 or 3.5 ng of cDNA in a total volume of 25 µl. Reactions were run in triplicates. The relative expression ratio was calculated according to Pfaffl [64] using the primary sigma factor *rpoD* (*BCAM0918*) as housekeeping gene. The primers used are listed in Table S4.

### Statistical analyses

Continuous normally distributed data were analyzed by using an independent sample t-test. P-values were determined using SPSS software, version 21.0. The over-representation analysis of EggNOG functional categories was carried out using Fisher's Exact tests.

## Supporting Information

Figure S1 **Decreased siderophore production in micro-oxic conditions.** Siderophore production of *B. cenocepacia* H111 grown under aerobic (black bar) and micro-oxic (grey bar) conditions was measured on CAS plates. The measured halo diameter corresponds to siderophore activity. Whiskers indicate SD, n = 3.
(TIF)

Figure S2 **Validation of four micro-oxic induced genes by** ***lacZ*** **fusions.** The activity of BCAL2780 (thioredoxin domain containing protein), BCAM1259 (sigma factor), *wcaJ* (CCE50896, sugar transferase in cepacian cluster II), *bclA* (lectin) and *cepI* promoter fusion was determined in the wild type grown in aerobic (black bar) and micro-oxic (grey bar) conditions. Whiskers indicate SD, n = 3.
(TIF)

Figure S3 **Proteins identified by the exclusion list approach add 272 preferentially low abundant proteins.** The exclusion list approach was successful in adding preferentially lower abundant proteins (red curve) on top of those identified over all discovery runs (blue curve) and allowed us to dig deeper into the proteome. For calculation of the relative protein abundance, see Methods.
(TIF)

Table S1 **Shotgun proteomics and RNA-Seq data for all** ***B. cenocepacia*** **H111 genes/proteins grown in micro-oxic (M) conditions and aerobic (A) conditions.**
(XLSX)

Table S2 **List of 55** ***B. cenocepacia*** **H111 and J2315 genes that are commonly induced by low oxygen (M) (DESeq analysis, p-value<0.2 for H111, fold change >2 for J2315). The expression in aerobic cells was taken as baseline (A).**
(XLSX)

Table S3 **Q-PCR results for selected genes.**
(XLSX)

Table S4 **Bacterial strains, plasmids and oligonucleotides used in this study.**
(DOCX)
