# Supplementary figures and images for: Response of Burkholderia cenocepacia H111 to Micro-Oxia
Source: PLoS One. 2013 Sep 2;8(9):e72939. doi: 10.1371/journal.pone.0072939 (PMC3759415; doi:10.1371/journal.pone.0072939)

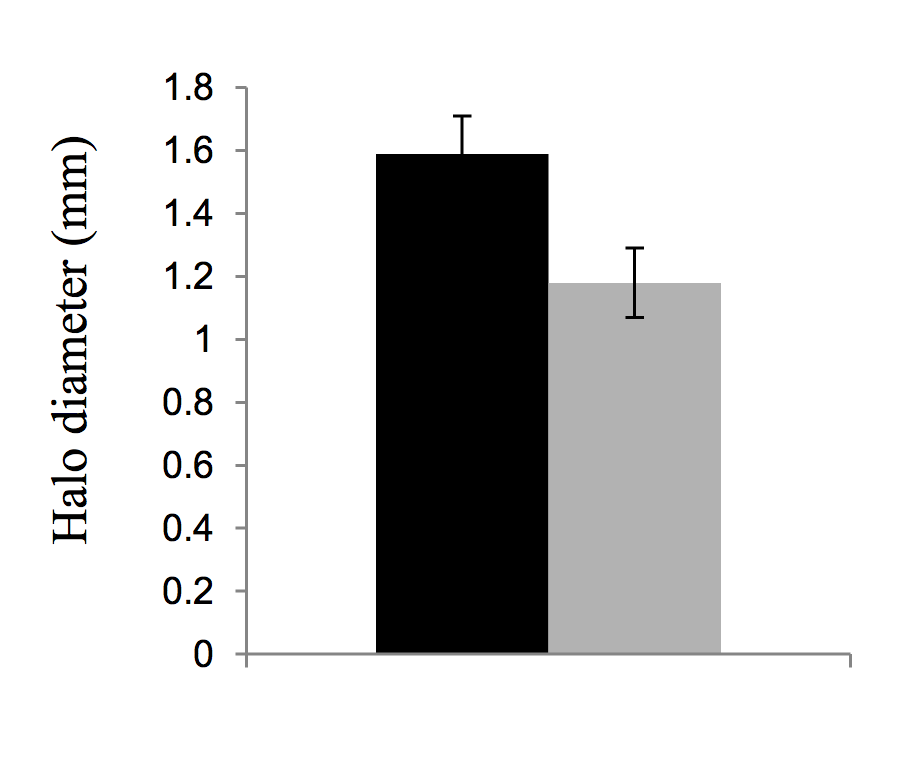

Supplement: Figure S1 — Decreased siderophore production in micro-oxic conditions. Siderophore production of B. cenocepacia H111 grown under aerobic (black bar) and micro-oxic (grey bar) conditions was measured on CAS plates. The measured halo diameter corresponds to siderophore activity. Whiskers indicate SD, n = 3. (TIF) [file pone.0072939.s001.tif]

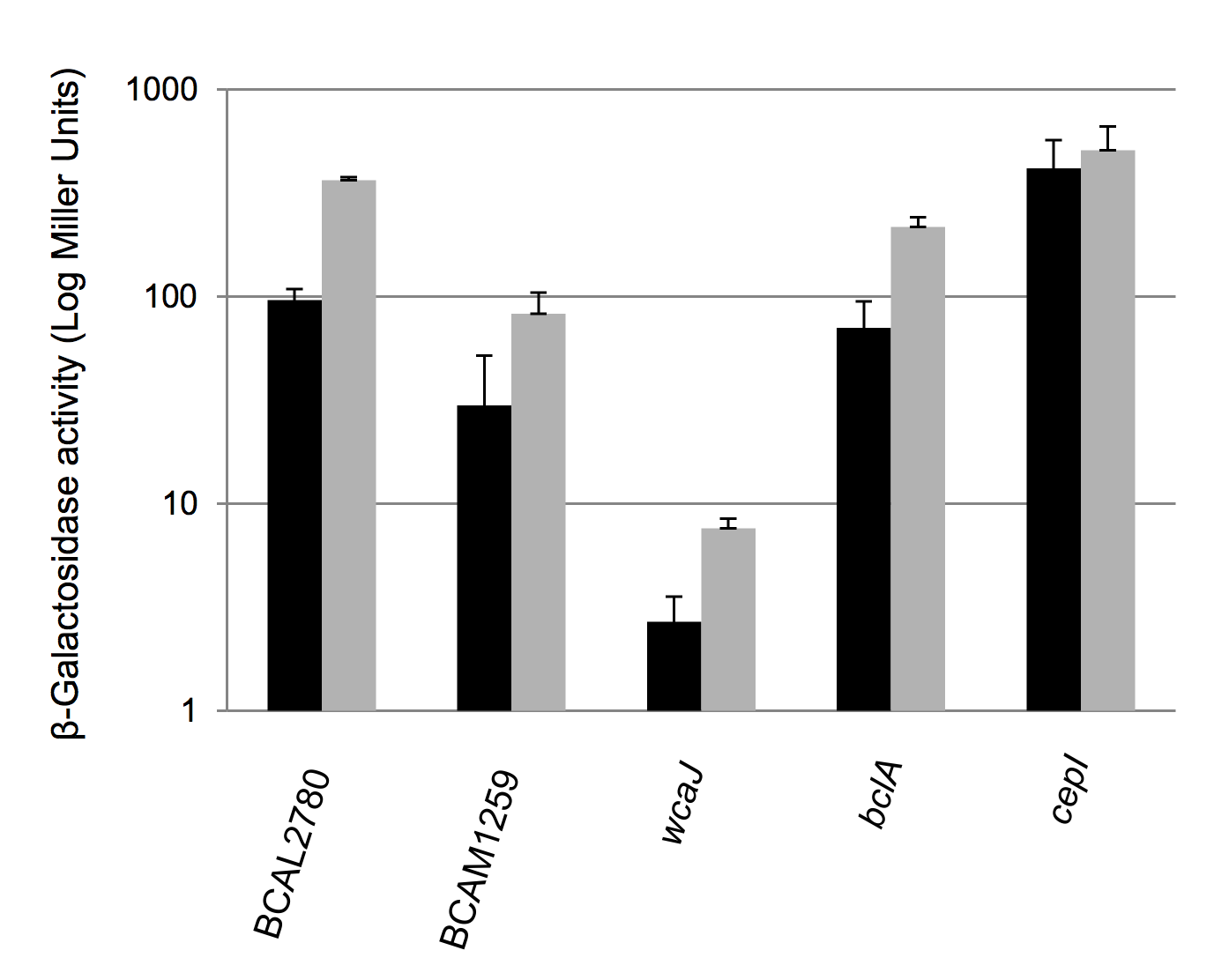

Supplement: Figure S2 — Validation of four micro-oxic induced genes by lacZ fusions. The activity of BCAL2780 (thioredoxin domain containing protein), BCAM1259 (sigma factor), wcaJ (CCE50896, sugar transferase in cepacian cluster II), bclA (lectin) and cepI promoter fusion was determined in the wild type grown in aerobic (black bar) and micro-oxic (grey bar) conditions. Whiskers indicate SD, n = 3. (TIF) [file pone.0072939.s002.tif]

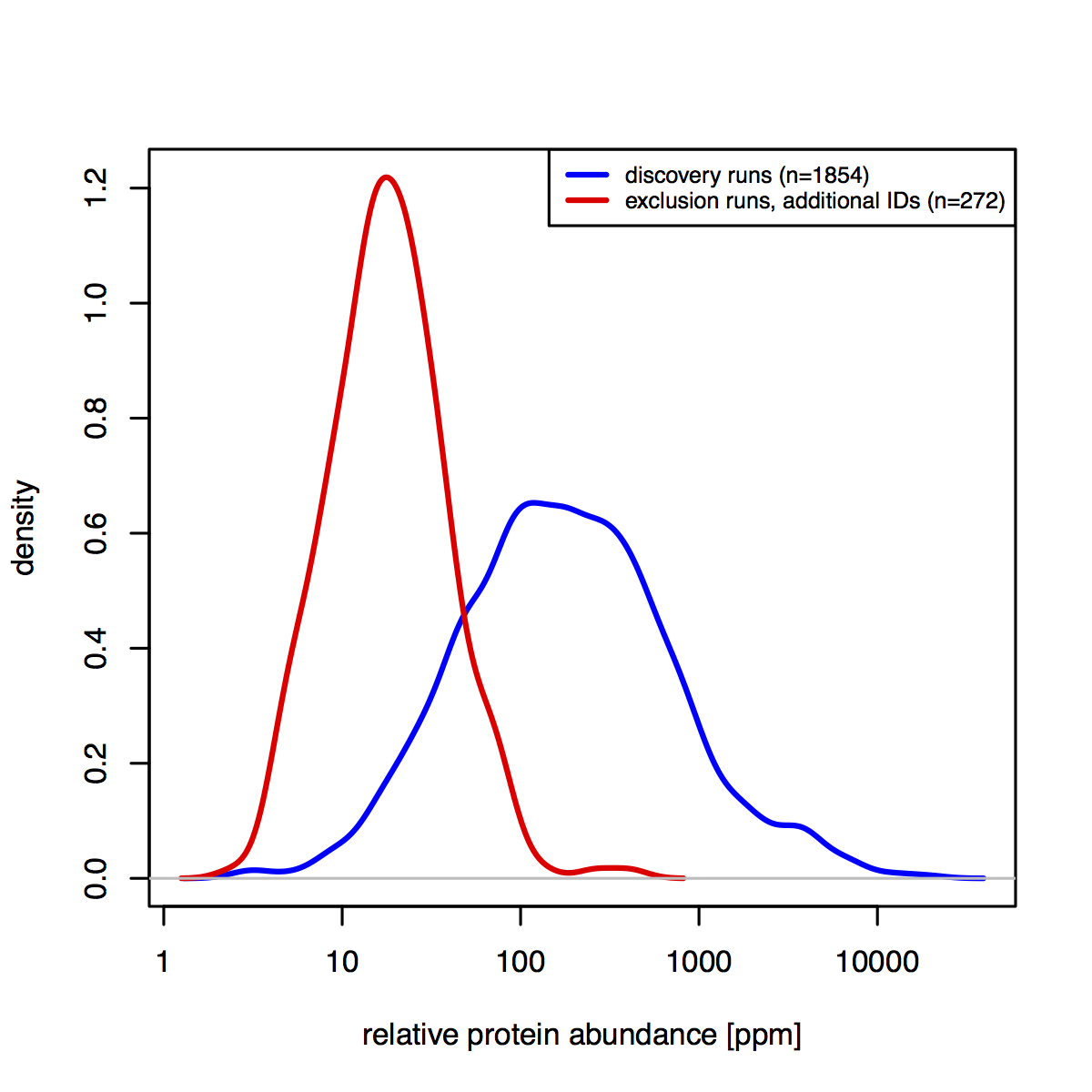

Supplement: Figure S3 — Proteins identified by the exclusion list approach add 272 preferentially low abundant proteins. The exclusion list approach was successful in adding preferentially lower abundant proteins (red curve) on top of those identified over all discovery runs (blue curve) and allowed us to dig deeper into the proteome. For calculation of the relative protein abundance, see Methods. (TIF) [file pone.0072939.s003.tif]
