# Supplementary material for: Response of Burkholderia cenocepacia H111 to Micro-Oxia
Source: PLoS One. 2013 Sep 2;8(9):e72939. doi: 10.1371/journal.pone.0072939 (PMC3759415; doi:10.1371/journal.pone.0072939)
Supplement: Table S4 — Bacterial strains, plasmids and oligonucleotides used in this study. (DOCX) [file pone.0072939.s007.docx]

# **Table S4:** Bacterial strains, plasmids and oligonucleotides used in this study.

| **Strain or plasmid** | **Description** | **Reference** |
| --- | --- | --- |
| Strains |  |  |
| *E. coli* |  |  |
| DH5α | F^-^ Φ80*lacZ*∆M15 ∆(*lacZYA-argF*) *recA1 endA gyrA96 thi-1 hsdR17 supE44 relAl deoR(U169)* | [1] |
| Top10 | Δ*lacX74 ara*Δ*139*Δ*(ara-leu)* | Invitrogen |
| *B. cenocepacia* |  |  |
| H111 | CF isolate from Germany, genomovar III | [2,3] |
| Plasmids |  |  |
| pSU11 | promoter probe vector; Gm^R^ | [4] |
| pSU11Tp | pSU11 derivative harboring dhfr cassette from pRN3, Tp^R^ | [5] |
| pP*_bclA_*-*lacZ* | pSU11 containing the *bclA* promoter region | [6] |
| pP*_cepI_*-*lacZ* | pSU11 containing the *cepI* promoter region | [5] |
| pP*_BCAL2780_*-*lacZ* | pSU11 containing the *BCAL2780* promoter region | This study |
| pP*_BCAM1259_*-*lacZ* | pSU11 containing the *BCAM1259* promoter region | This study |
| pP*_wcaJ-_lacZ* | pSU11 containing the *CCE50896* promoter region | This study |
| Oligonucleotides^a^ |  |  |
| BCAM0918_F | AGGAAGCCGAAGAGGAAGAG | This study |
| BCAM0918_R | GTCGAACCACTCGCTGATCT | This study |
| BCAM0049_F | GTCTGCGTGATTCCGTACA | This study |
| BCAM0049_R | CTGTTCGCTCATCAGCTTGT | This study |
| BCAM1259_F | CGCGCAAGTACCTGAACC | This study |
| BCAM1259_R | GAATCCACCAGGTCGCATAG | This study |
| BCAL0785_F | TCGACCAGCACAAGCAGTAT | This study |
| BCAL0785_R | ATGATCCGGAACGAGAAGAA | This study |
| BCAL1919_F | GCAGCTCAAGGAAGAGATCG | This study |
| BCAL1919_R | GGTTGTGCTGTTCCTGCTCT | This study |
| BCAM1010_F | CTACGTATTCATGCCGACGA | This study |
| BCAM1010_R | GTAGTAGCGATACGCGAGCA | This study |
| BCAL2118_F | GACCTGATCTGGTGCGAAAC | This study |
| BCAL2118_R | CGTCGTCGAGGTTCTTCTTC | This study |
| *wcaJ*_CCE50896 _F | AAGCTTGGAAAGAACACGACGACCAG | This study |
| *wcaJ*_CCE50896 _R | CTCGAGGATGACACTCTCCACGGTTG | This study |
| BCAM1259_lacZ_F | CTCGAGGAGTAGTACGCCGGGTAAGC | This study |
| BCAM1259_lacZ_R | AAGCTTGGCGTAGTCATGTCGATGG | This study |
| BCAL2780_lacZ_F | CTCGAGGAACGCTACATCGAGTGGAA | This study |
| BCAL2780_lacZ_R | CTCGAGTCCGTTCGAATATCGTCAGG | This study |

# References

1. Hanahan D (1983) Studies on transformation of *Escherichia coli* with plasmids. Journal of Molecular Biology 166: 557–580. doi:10.1016/S0022-2836(83)80284-8.

2. Römling U, Wingender J, Müller H, Tümmler B (1994) A major *Pseudomonas aeruginosa* clone common to patients and aquatic habitats. Appl Environ Microbiol 60: 1734–1738.

3. Gotschlich A, Huber B, Geisenberger O, Tögl A, Steidle A, et al. (2001) Synthesis of multiple *N*-acylhomoserine lactones is wide-spread among the members of the *Burkholderia cepacia* complex. Syst Appl Microbiol 24: 1–14. doi:10.1078/0723-2020-00013.

4. O’Grady EP, Viteri DF, Malott RJ, Sokol PA (2009) Reciprocal regulation by the CepIR and CciIR quorum sensing systems in *Burkholderia cenocepacia*. BMC Genomics 10: 441. doi:10.1186/1471-2164-10-441.

5. Schmid N, Pessi G, Deng Y, Aguilar C, Carlier AL, et al. (2012) The AHL- and BDSF-dependent quorum sensing systems control specific and overlapping sets of genes in *Burkholderia cenocepacia* H111. PLoS One 7: e49966.

6. Inhülsen S, Aguilar C, Schmid N, Suppiger A, Riedel K, et al. (2012) Identification of functions linking quorum sensing with biofilm formation in *Burkholderia cenocepacia* H111. MicrobiologyOpen 1: 225–242. doi:10.1002/mbo3.24.
